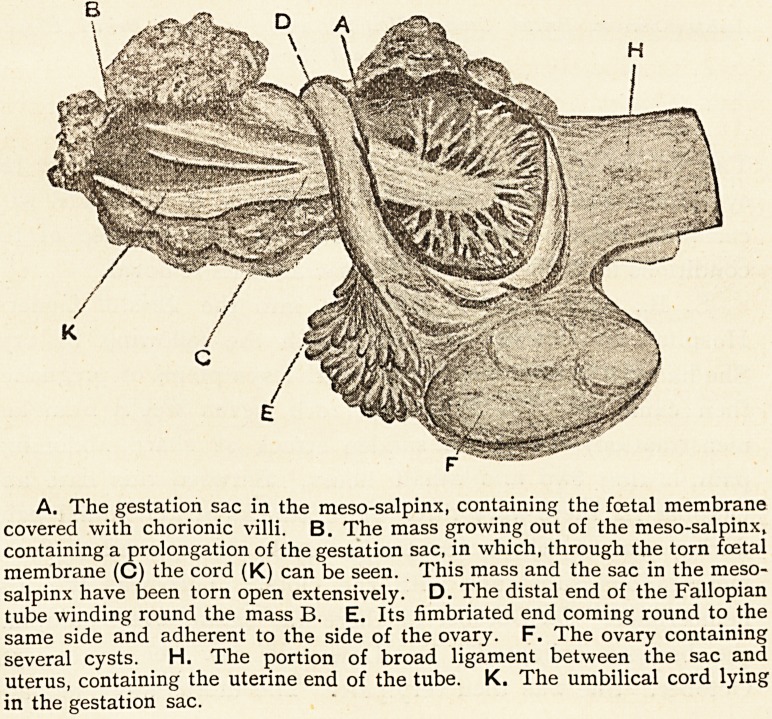# A Case of Extra-Uterine Gestation

**Published:** 1893-03

**Authors:** A. E. Aust Lawrence, Charles A. Morton

**Affiliations:** Physician Accoucheur to the Bristol General Hospital, Lecturer on Midwifery and Diseases of Women at the Bristol Medical School; Assistant-Surgeon Bristol General Hospital, and Pathologist Bristol Hospital for Children and Women


### A CASE OF EXTRA-UTERINE GESTATION.

BY

A. E. AUST LAWRENCE, M.D. Aberd.,

*Physician Accoucheur to the Bristol General Hospital, Lecturer on Midwifery and Diseases of Women at the Bristol Medical School,*

AND

CHARLES A. MORTON, F.R.C.S. Eng.,

*Assistant-Surgeon Bristol General Hospital, and Pathologist Bristol Hospital for Children and Women.*

---

THE following case is worthy of record, not only because the symptoms and signs enabled a diagnosis to be made with certainty before abdominal section, but also because of the conditions found in the gestation sac after its removal.

S. B., aged 26, was admitted into the Bristol General Hospital, on October 4th, 1892, with the following history: She had last menstruated on July 4th; symptoms of pregnancy then came on. On September 10th (seven weeks from last menstruation) she had a sudden attack of sharp abdominal pain, lasting two and a-half hours. Between this date and October 1st there were three more attacks of abdominal pain, during one of which (September 27th) she passed a fleshy membranous substance with the clots. This was the first hemorrhage. On October 1st there was again severe pain, and she became collapsed. Dr. Lawrence saw her on the 5th October. She was then very pale. The uterus was fixed, and there was a swelling to the right side and in front, extending up into the right groin and above the pubic symphysis. The abdomen was opened on the 7th. The gestation sac was found very adherent to the pelvic brim. No embryo was found. The broad ligament between the sac and the uterus was tied, and the whole sac removed. The peritoneum was drained for some time, and she recovered.

After much prolonged and careful examination of the parts removed, the following condition was found: The gestation sac was contained in the layers of the broad ligament above the ovary (*i.e.* in the meso-salpinx) and in a mass of soft tissue which had grown out from the meso-salpinx. The distal end of the Fallopian tube wound round this outgrowing mass, the fimbriated end being adherent to the ovary. The gestation sac was extensively ruptured, in both the broad ligament and this outgrowth of tissue, probably partly in its removal, as it had to be torn away from its adhesions. The outgrowth would not fit

A. The gestation sac in the meso-salpinx, containing the fœtal membrane covered with chorionic villi. B. The mass growing out of the meso-salpinx, containing a prolongation of the gestation sac, in which, through the torn fœtal membrane (C) the cord (K) can be seen. This mass and the sac in the meso-salpinx have been torn open extensively. D. The distal end of the Fallopian tube winding round the mass B. E. Its fimbriated end coming round to the same side and adherent to the side of the ovary. F. The ovary containing several cysts. H. The portion of broad ligament between the sac and uterus, containing the uterine end of the tube. K. The umbilical cord lying in the gestation sac.

on to the broad ligament, as the tube curled over between (nor could it be passed under the tube), and was not therefore a mass partly broken off from the broad ligament. As the fœtal membrane passed from the mass outside into the cavity in the meso-salpinx, it became covered with chorionic villi, and these villi were embedded in the tissue at the uterine side of the ligament part of the sac. The ruptured cord was found in the gestation sac. About an inch of the uterine end of the Fallopian tube was found, the distal part of which was blocked by placental growth. The middle portion of the tube was absent, and had probably formed the roof of the gestation sac which was so extensively torn. The distal portion of the tube passing from the gestation sac wound round the projecting mass of tissue already described. The lumen of this portion of the tube was quite patent, and so was the abdominal ostium. Mr. Bland Sutton's investigations have shown that if the gestation remains in the tube after the eighth week the abdominal ostium is closed;[1] hence, in this case (as no force was used in passing a probe through the ostium), the gestation sac must have ruptured into the layers of the broad ligament before the eighth week. This was, of course, the "primary rupture," and may have taken place during the attack of severe pain and vomiting on September 7th, though the passage of the gestation between the layers of the broad ligament is a gradual process. However, the sudden distension by hemorrhage may have caused the symptoms. The gestation then continued to develop in the meso-salpinx, and eventually projected from it, finally rupturing into the peritoneum.

When the specimen was shown at the Bristol Medico-Chirurgical Society[2] an objection was raised to this description of the pathological condition. It was said that when once the gestation sac ruptures out of the tube into the broad ligament the gestation either perishes or grows to full time, or nearly so, and that secondary rupture at the time stated did not occur. But this condition is clearly described by Mr. Bland Sutton, and

[1] *Surgical Diseases of the Ovaries and Fallopian Tubes, including Tubal Pregnancy*, 1891, p. 312.

[2] The specimen was shown also at the Obstetrical Society.

is said by him to occur about the time when it happened in this case.[1] Moreover, the condition of the abdominal ostium, as before described, proves it to have passed from the tube into the broad ligament before the eighth week, and the nature of the gestation sac negatives the supposition that it remained wholly as a tubal pregnancy.

A microscopic examination of the mucous membrane of the uterine end of the tube was made, but no evidence could be discovered of any desquamative salpingitis, such as Mr. Lawson Tait supposes to be a possible cause of the development of the embryo in the tube.

---

## Figures and Tables

**Figure f1:**